# Intra-articular delivery of Si-Vangl2 limits cartilage degeneration in an osteoarthritis rat model

**DOI:** 10.3389/fbioe.2026.1773841

**Published:** 2026-03-30

**Authors:** Peiru Li, Ke Zhang, Zhuoying Li, Yunyang Lu, Hong Zhang

**Affiliations:** 1 Hospital of Stomatology, Sun Yat-sen University, Guangdong Provincial Key Laboratory of Stomatology, Guanghua School of Stomatology, Sun Yat-sen University, Guangzhou, Guangdong, China; 2 Department of Stomatology, Longgang Central Hospital of Shenzhen, Shenzhen, Guangdong, China

**Keywords:** cartilage degeneration, extracellular matrix, hypertrophy, JNK, NF-κB, osteoarthritis, PCP, Vang2

## Abstract

Osteoarthritis (OA) is driven by progressive cartilage degeneration associated with dysregulated chondrocyte metabolism and inflammatory signaling, for which effective disease-modifying treatments remain limited. In this study, we observed that Van Gogh-like 2 (Vangl2), a core component of the non-canonical Wnt/planar cell polarity (PCP) pathway, is upregulated in chondrocytes under inflammatory conditions. In a collagenase-induced OA rat model, intra-articular delivery of small interfering RNA targeting Vangl2 (Si-Vangl2) attenuated cartilage degeneration and partially preserved cartilage structure and matrix. Immunohistochemical (IHC) analysis showed that Vangl2 silencing increased the expression of anabolic and proliferative markers including Sox9, type II collagen (Col2), aggrecan, and PCNA, while reducing the levels of catabolic enzymes including matrix metalloproteinase 3 (MMP3), MMP13, as well as the hypertrophic marker type X collagen (Col10a1). Wnt5a-associated inflammatory signaling through JNK and NF-κB pathways, was also diminished following Si-Vangl2 treatment. *In vitro* findings suggest that Vangl2 act as a pro-chondrogenic factor, examined by real-time quantitative polymerase chain reaction (RT-qPCR), Western blot and cell counting kit-8 (CCK-8) assays. These results indicate that Si-Vangl2 limits cartilage degeneration by enhancing anabolic activity, suppressing matrix degradation and chondrocyte hypertrophy, and restoring chondrocyte homeostasis in OA.

## Introduction

Osteoarthritis (OA) is one of the most prevalent degenerative joint disorders worldwide and represents a major public health challenge due to chronic pain and disability in the aging population (Martel-Pelletier et al., 2016; Loeser et al., 2016). In 2021, knee OA affected more than 500 million individuals globally, accounting for approximately 22% of adults older than 40 years (Quicke et al., 2022). Histologically, OA is defined by progressive articular cartilage degradation, subchondral bone remodeling, and synovial inflammation. Among these pathological changes, articular cartilage destruction is considered the hallmark of most OA cases (Loeser et al., 2016; Yao et al., 2023). Chondrocytes, the sole resident cell type within cartilage, undergo profound phenotypic dysregulation during the OA progression. These alternations are manifested by higher rates of cell death, enhanced secretion of extracellular matrix (ECM)-degrading enzymes such as MMPs, and abnormal hypertrophic differentiation (van der Kraan and van den Berg, 2012; Guan et al., 2024). Despite the high prevalence and burden of OA, current treatments mainly offer symptomatic relief, and effective strategies to limit cartilage degeneration remain insufficient.

However, a recent study demonstrates that cartilage regeneration can be achieved through phenotypic reprogramming of resident mature chondrocytes. Rather than relying on stem or progenitor cell proliferation, redirecting hypertrophic or catabolic chondrocytes toward a matrix-synthesizing phenotype has emerged as a potential endogenous repair strategy (Singla et al., 2025). This concept highlights the therapeutic potential of targeting key genes within resident chondrocytes to restore anabolic-catabolic balance and maintain cartilage homeostasis.

Growing evidence implicates non-canonical Wnt signaling, particularly the planar cell polarity (PCP) pathway, in the pathogenesis of OA. Activation of the Wnt/PCP pathway has been shown to regulate chondrocyte metabolism, enhance ECM degradation, and alter subchondral bone remodeling (Sassi et al., 2014; Zhang et al., 2014; Shi et al., 2016; Tong et al., 2019; De Palma and Nalesso, 2021; Gill et al., 2023; Tonutti et al., 2023; Minghua et al., 2024). As excessive inhibition of canonical Wnt/β-catenin signaling has been shown to disrupt growth plate chondrocyte proliferation (Chen et al., 2008), alternative β-catenin independent Wnt pathway-related strategies warrant further investigation (Yao et al., 2023). Thus, the therapeutics targeting of the Wnt/PCP pathway in OA have garnered growing interest (Fan et al., 2024; Tong et al., 2019; Huang et al., 2017; Thorup et al., 2020; Wang et al., 2019). Studies on human OA tissues have demonstrated that the non-canonical Wnt/PCP pathway ligand Wnt5a is markedly upregulated in both chondrocytes and osteoblasts (Huang et al., 2017; Martineau et al., 2017). Wnt5a is associated with the increased expression of MMPs and pro-inflammatory mediators by downstream PCP or Wnt/Ca^2+^ pathways through the activation of MAPK and NF-κB signaling, thereby inducing cartilage catabolism and joint degeneration (Zhang et al., 2014; 2021; Huang et al., 2017; Martineau et al., 2017).

Vangl2, a core PCP component, plays a crucial role in cell polarity, cytoskeletal organization, and chondrocyte orientation during endochondral osteogenesis and limb elongation (Song et al., 2010; Gao et al., 2011; Cheong et al., 2020; Li et al., 2023). Our previous *in vitro* study demonstrated that silencing Vangl2 markedly attenuated the expression of proinflammatory cytokines and matrix-degrading enzymes in ATDC5 progenitor-like chondrogenic cell line, thereby alleviating inflammatory and catabolic responses (Zhang et al., 2021). However, whether intra-articular silencing of Vangl2 can preserve cartilage integrity and restore chondrocyte anabolic–catabolic balance *in vivo* during OA progression remains unclear.

Therefore, the present study aimed to evaluate the therapeutic efficacy of intra-articular Vangl2 silencing in experimental OA model and to elucidate its role in regulating chondrocyte anabolic activity, matrix catabolism, hypertrophic differentiation, and associated inflammatory signaling pathways.

## Materials and methods

### Study design

This study comprised both *in vitro* and *in vivo* experiments. *In vitro*, the ATDC5 chondrocyte cell line was used to investigate the expression pattern of Vangl2 under inflammatory stimulation and during chondrogenic differentiation. Cells were cultured and treated with interleukin-1β (IL-1β) to mimic an inflammatory microenvironment. Vangl2 expression and other PCP-related genes were quantified by RT-qPCR. Cell proliferation was assessed using the CCK-8 assay, and the effects of Vangl2 overexpression or silencing were further evaluated by accessing Sox9 and BMP2 protein levels via Western blotting.

*In vivo*, rats were assigned to groups to exam the therapeutic effect of Si-Vangl2 administration in CIA model. All protocols were approved by the Ethics Committee of the Institution Animal Care and Use Committee, Sun Yat-sen University. (Approval NO.: SYSU-IACUC-2022–000191). At the end of treatment, rats were euthanized and the knee joints were harvested for histological and immunohistochemical analyses. Histological assessments included hematoxylin–eosin (H&E) and Safranin O/Fast Green. IHC staining was performed to evaluate chondrocyte proliferation, differentiation, inflammatory response, and hypertrophy. All sample preparation, staining, and scoring were performed by investigators blinded to group allocation.

### ATDC5 cell culture and differentiation

Progenitor chondrogenic cell lines ATDC5 (Fuheng Centre Cell Bank, China) were cultured in Dulbecco’s Modified Eagle’s Medium/Ham’s F-12 nutrient mixture (DMEM/F12; Gibco, United States) supplemented with 10% fetal bovine serum (FBS; Gibco, United States) and 1% penicillin–streptomycin (Gibco, United States). To induce chondrogenic differentiation, 1% Insulin-Transferrin-Selenium solution (ITS; Gibco, United States) were added to the differentiation medium. Cells were maintained at 37 °C in a humidified atmosphere containing 5% CO_2_. Differentiation was maintained for 21 days, and the medium was refreshed every 2 days.

During the differentiation period, cells were harvested at 3-day intervals (Day 0, 3, 6, 9, 12, 15, 18, and 21). At each time point, glycosaminoglycan deposition was evaluated by Alcian Blue staining, and total RNA was extracted for quantitative RT-PCR analysis of Vangl2 expression. This time-course design allowed dynamic assessment of extracellular matrix production and Vangl2 transcriptional changes during chondrogenic differentiation.

### RNA isolation and RT-qPCR

To simulate an inflammatory microenvironment, ATDC5 cells were treated with recombinant mouse interleukin-1β (IL-1β; 10 ng/mL; PeproTech, United States) for 24 h.

Total RNA was extracted using TRIzol reagent (Invitrogen, United States) following the manufacturer’s protocol. RNA was reverse-transcribed into cDNA using the PrimeScript™ RT Master Mix (Perfect Real Time; Takara, Japan). Quantitative PCR was performed using a SYBR® Green RT-qPCR kit (Roche Diagnostics, Switzerland) on a Roche LightCycler 96 instrument.

The primer sequences used for amplification are listed in Table 1. The mRNA expression levels of target genes were normalized to GAPDH expression, and relative expression was calculated using the 2^−^ΔΔCt method. Each reaction was performed in triplicate, and the control group was assigned an arbitrary value of 1 for baseline normalization. Treated samples were expressed as fold change relative to control.

**TABLE 1:** Primer sequences for PCR.

Gene (mouse)	Primer sequence (5'→3′)forward	Primer sequence (3'→5′)reverse
Wnt5a	CGCTGCTGGAGTGGTAA	GTC​CCG​AGG​TAA​GTC​CTT​G
Ror2	TGC​CAC​TTC​GTC​TTT​CCT​C	GGC​ACA​GGT​CAT​TCT​CCA​A
Ryk	GTC​ACT​ACG​CTC​TGT​CCT​TTA​AC	GCT​CGA​CCC​GAA​ACA​CTG​ATA​A
Vangl1	TTA​GCA​AGG​ACA​TGG​AGG​A	TAGCAGGATGAAGGCGAT
Vangl2	TCTCGTCCCCTGCCTTA	TGGTCCCTCACCCTGAT
Prickle1	CTTCTGTCTCCCGCTCTG	CCT​GAT​CCA​CTC​TGG​CTT​T
Prickle2	TTG​GCT​TCT​AGT​TGT​TTG​TCT​GT	GGG​ACC​CAG​GCG​TAC​TCT​T
Dvl1	TCTGTACCCTGGCCCTTG	TGC​TCT​TGC​TCC​CTT​CAC​T
Dvl2	GGGCAACCCCAGTGAGT	GGGCAACCCCAGTGAGT
Dvl3	GCCCCTTTCTGTGCTGAC	TGT​CCA​AGT​TCT​CCT​GGC​T
Celsr1	TCG​CTG​ACT​TCG​GTG​CTT​G	TTA​CCA​GCT​CTA​CCC​AAA​CGG
Celsr2	AAA​TGC​TGC​ATG​AAC​CGT​TTT​T	CTG​CGC​TTA​CCC​TTC​CTC​C
Celsr3	CCC​GGT​ACT​ACT​GCT​CCT​TCT	GAC​AAA​GAG​CTA​CGG​CTC​CA

### Cell transfection

ATDC5 cells were seeded in six-well plates (Corning, NY, United States) at a density of 5 × 10^5^ cells/well. When cells reached 50%–70% confluence, small interfering RNA targeting Vangl2 (Si-Vangl2; RiboBio, Guangzhou, China) or a negative control siRNA (Si-NC; RiboBio, Guangzhou, China) was transfected using Lipofectamine RNAiMAX reagent (Invitrogen, CA, United States) according to the manufacturer’s instructions.

For overexpression assays, cells were transfected with a Vangl2 expression plasmid or a negative control vector (NC) using Lipofectamine 2000 (Invitrogen, CA, United States) when cell confluence reached approximately 70%. After 6 h, the medium was replaced with fresh DMEM/F12 containing 10% FBS, and cells were further incubated for an additional 48 h before analysis. Cells were then collected for Western blot and cell proliferation assay.

### Western blot analysis

Total proteins were extracted using RIPA lysis bufferr (Beyotime Institute of Biotechnology, China) containing 1% protease and phosphatase inhibitors (Beyotime, China). Protein concentrations were determined by BCA assay (Thermo Fisher Scientific). Protein samples were added to the loading buffer (loading buffer to sample ratio, 1:4) and were heated to 99 °C for 10 min to denature the proteins. Sodium dodecyl sulfate-polyacrylamide gel electrophoresis (Beyotime, China) was used to separate proteins. After transferring the proteins to polyvinylidene fluoride membranes (Millipore, United States) and blocking with 5% non-fat milk for 1 h at room temperature, membranes were incubated overnight at 4 °C with primary antibodies against Vangl2 (1:800), Sox9 (1:2000), BMP2(1:2000) and GAPDH (1:3000) (Affinity Biosciences, United States). After washing, membranes were incubated with a horseradish peroxide-conjugated goat anti-rabbit or anti-mouse IgG (1:2000; Cell Signaling Technology, United States) for 1 h (RT). Protein bands were visualized using ECL kit (Millipore, United States) and quantified with ImageJ software.

### Cell proliferation assay

Cell proliferation was assessed using the Cell Counting Kit-8 (CCK-8; OONIE, China). After transfection, ATDC5 cells of four groups as mentioned were harvested, counted with a hemocytometer, and seeded into 96-well plates at a density of 1.5 × 10^3^ cells/well (five replicates per group, 100 μL medium per well). PBS was added to the peripheral wells to prevent evaporation. After 8 h of incubation for cell attachment, one plate was used as the 0 h time point. The medium was replaced with 10% CCK-8 solution, followed by incubation for 1 h at 37 °C in the dark. Absorbance was measured at 450 nm using a microplate reader (Bio-Rad, United States). The same procedure was repeated at 24, 48, and 72 h to record cell viability and generate growth curves.

### OA induction and intra-articular administration of Si-RNA

Twenty male Sprague–Dawley rats (8 weeks old, 250–280 g) were obtained from Animal Experiment Center of Sun Yat-sen University. All protocols were approved by the Ethics Committee of the Institution Animal Care and Use Committee, Sun Yat-sen University (Approval NO.: SYSU-IACUC-2022–000191). Rats were housed under specific pathogen-free (SPF) conditions with free access to food and water. Animals were randomly divided into four groups (n = 5 per group).

1. Control group (saline injection),
2. CIA group (OA model),
3. Si-nc + CIA group (negative control),
4. Si-Vangl2 + CIA group (treatment).

All intra-articular injections were performed under general anesthesia induced by intraperitoneal injection of pentobarbital sodium (30 mg/kg body weight).

OA was induced by intra-articular injection of 100 µL collagenase type II (Gibco, America) (20 mg/mL) into the right knee joint on day 0, while same dose of saline was injected into the same portion of rats in control group. Injections were performed through the lateral patellar tendon, with correct joint cavity entry confirmed by a loss of resistance.

After model confirmation on day 7, intra-articular treatments were initiated. Rats in the Si-Vangl2 + CIA and Si-nc + CIA groups respectively received the Vangl2 small interfering RNA (siRNA; 100µL, 0.2 nmol/ml; RiboBio; China) and negative control (nc; 100µL, 0.2 nmol/ml; RiboBio; China) at right knee once per week for 4 weeks. Control and CIA groups received equivalent volumes of saline.

### Histology

At the end of the treatment, rats were euthanized by intraperitoneal overdose of pentobarbital sodium (200 mg/kg body weight), followed by confirmation of death prior to tissue collection. The right knee joints were collected with the excess muscle tissue removed, fixed in 4% paraformaldehyde for 48 h, decalcified in 10% EDTA for 12 weeks, and then dehydrated and embedded in paraffin. Paraffin-embedded knee joints were cut into 4 µm sections. Hematoxylin–eosin (H-E), Safranin O/Fast Green (S-O), and Alcian Blue were performed following standard protocols to evaluate cartilage morphology and proteoglycan content. Cartilage degeneration was evaluated by the Osteoarthritis Research Society International (OARSI) scoring system by two blinded observers.

Immunohistochemical analysis was conducted on sections of rat knee joints with primary antibodies against Vangl2, Wnt5a, Sox9, Type II collagen, Aggrecan, PCNA, MMP3, MMP13, Type X collagen, phospho-JNK, and phospho-p65 (Affinity Biosciences, United States), followed by HRP-conjugated secondary antibodies and DAB development. The stained sections were observed under a fluorescence microscope (Leica Aperio AT2, Germany). DAB staining intensity was quantified using ImageJ software with uniform global thresholding applied to each antibody set. Representative images and the results of semi-quantitative analysis derived from at least three replicates are presented.

### Statistical analysis

All data were presented as mean ± standard deviation (SD) from at least three independent experiments. Statistical analysis was performed using GraphPad Prism 10.0. Differences among multiple groups were analyzed using one-way analysis of variance (ANOVA) followed by Turkey’s *post hoc* test. A *P* value <0.05 was considered statistically significant.

## Results

### Screening of Wnt/PCP pathway genes under IL-1β–induced inflammation identified Vangl2 as the most upregulated gene in chondrocytes

**FIGURE 1:**
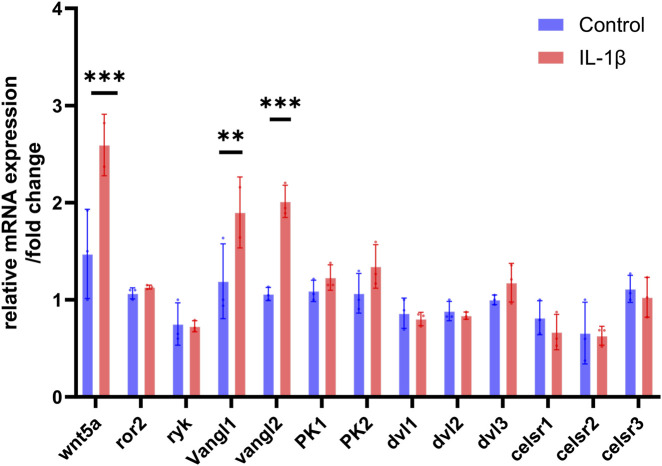
The expression of PCP-related gene in ATDC5 stimulated by IL-1β determined by RT-qPCR. The values are presented as means ± SD (n = 3). *p < 0.05, **p < 0.01, ***p < 0.001.

To examine the involvement of the Wnt/PCP pathway in inflammation-driven chondrocyte pathology, we analyzed the transcriptional profile of key PCP genes in ATDC5 cells stimulated with IL-1β. RT-qPCR revealed the activation of PCP signaling in ATDC5 cells under the IL-1β stimulation (Figure 1). Notably, Vangl2 exhibited a particularly marked elevation among the screened targets (P < 0.001). Wnt5a expression was also increased under inflammatory conditions. The simultaneous elevation of Wnt5a and Vangl2 in inflammatory chondrocytes indicates a potential involvement of Vangl2 in Wnt/PCP-associated OA changes.

### Vangl2 was involved in the proliferation and differentiation of ATDC5

**FIGURE 2:**
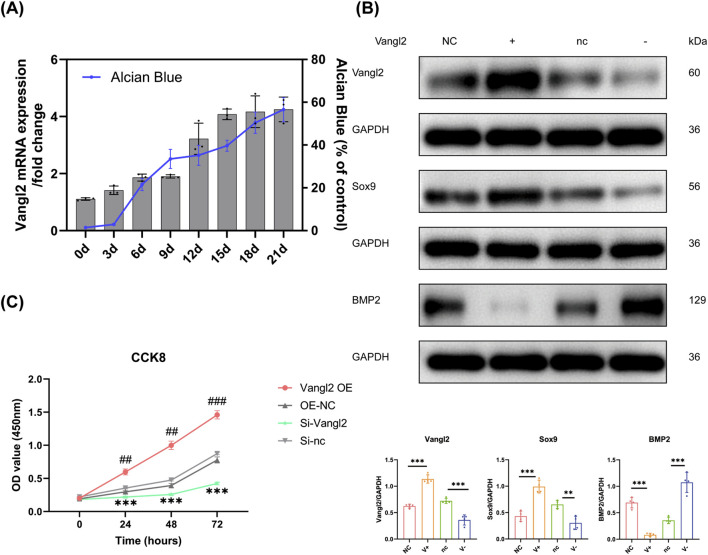
Vangl2 participates in proliferation and chondrogenic differentiation in ATDC5. **(A)** Expression trend of Vangl2 during chondrogenic differentiation indicated by RT-qPCR and its parallel increase with cartilage matrix deposition indicated by Alcian Blue staining intensity. The values are presented as means ± SD (n = 3). **(B)** Protein levels of Sox9 and BMP2 in ATDC5 cells 48 h after Vangl2 overexpression and knockdown detected by Western blotting. The values are presented as means ± SD (n = 4). **(C)** Cell proliferation assessed by CCK-8 assay following overexpression and knockdown of Vangl2 in ATDC5 cells, measured at 0,24,48 and 72 h. The values are presented as means ± SD (n = 3). #*p* < 0.05, ##*p* < 0.01, ###*p* < 0.001 vs. OE-NC. *p < 0.05, **p < 0.01, ***p < 0.001 vs. Si-nc.

Since inflammatory stimulation upregulates Vangl2 expression in ATDC5 cells, we further examined its expression pattern and functional relevance during chondrogenesis. Vangl2 mRNA levels increased during ATDC5 chondrogenic differentiation, particularly at late-stage differentiation and were accompanied by enhanced Alcian Blue staining intensity (Figure 2A).

ATDC5 cells were transfected with Vangl2 overexpression plasmid or Si-Vangl2, and protein levels were analyzed 48 h post-transfection. Western blot analysis showed increased Sox9 and reduced BMP2 levels following Vangl2 expression, with opposite changes after Vangl2 knockdown (Figure 2B). Cell proliferation was evaluated using CCK-8 assay after transfection (designated as 0 h), followed by measurements at 24 h, 48 h, and 72 h Vangl2 overexpression was associated with increased proliferative activity over time, whereas Vangl2 knockdown reduced proliferation compared with control groups (Figure 2C). Notably, although Vangl2 knockdown inhibited proliferation under normal conditions, it significantly rescued the IL-1β-induced suppression of ATDC5 cell proliferation (Supplementary Figure S1). These suggested that Vangl2 may participate in proliferation and differentiation associated processes during cartilage development and pathological remodeling.

### Intra-articular administration of Si-Vangl2 attenuated cartilage degeneration in a CIA rat model

**FIGURE 3:**
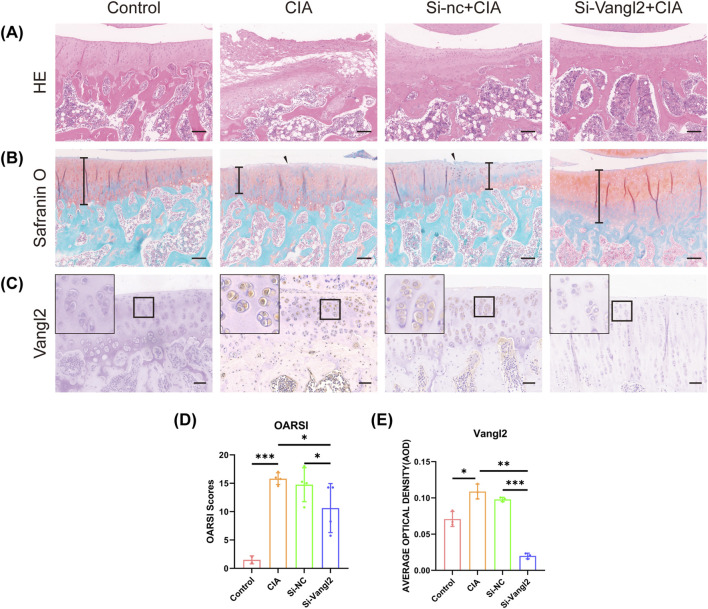
Intra-articular administration of Si-Vangl2 attenuates cartilage degeneration in a CIA rat model. **(A)** H-E staining of articular region of rats in the Control group, CIA group, Si-nc + CIA group and Si-Vangl2+CIA group. Scale bar = 150 μm. **(B)** S-O staining of articular region of rats in the Control group, CIA group, Si-nc + CIA group and Si-Vangl2+CIA group. **(C)** IHC staining of Vangl2 in articular region in the Control, CIA Si-NC + CIA and Si-Vangl2+CIA group. Scale bar = 50 μm. **(D)** OARSI scores in articular region of each group. Scale bar = 150 μm. The values are presented as means ± SD (n = 4). **(E)** The statistical analysis of staining of Vangl2 in articular region in each group. The values are presented as means ± SD (n = 3). *p < 0.05, **p < 0.01, ***p < 0.001.

H&E staining revealed severe cartilage surface disruption, fibrillation, matrix loss, cartilage thinning, and disorganized chondrocytes in the CIA and Si-nc + CIA groups. These degenerative changes were substantially improved by Si-Vangl2 treatment, which represented as smoother cartilage surfaces, increased cartilage thickness, and more organized chondrocyte arrangement (Figure 3A).

Safranin O–Fast Green staining showed a marked loss of matrix proteoglycans in the CIA and Si-nc + CIA groups. Si-Vangl2 treatment restored uniform and intense staining, indicating preserved cartilage matrix integrity (Figure 3B). Consistently, OARSI scores were reduced in the Si-Vangl2+CIA group compared with the CIA group (Figure 3D).

The therapeutic efficacy of Si-Vangl2 was evaluated in a CIA rat model. IHC analysis confirmed efficient Vangl2 knockdown in the articular cartilage following intra-articular administration of Si- Vangl2 (Figures 3C,E).

Subchondral bone remodeling in OA models was assessed via histological analysis, including H&E, Safranin O–Fast Green, and Alcian Blue staining. Histologically, the CIA group exhibited severe articular cartilage destruction, accompanied by disorganized and fragmented bone trabeculae, and increased marrow cavity vacuolization. Conversely, Si-Vangl2 administration promoted a more organized trabecular architecture and attenuated trabecular separation in the subchondral bone (Supplementary Figure S2).

### Si-Vangl2 rescued the expression of chondrogenic and proliferative markers in OA cartilage

**FIGURE 4:**
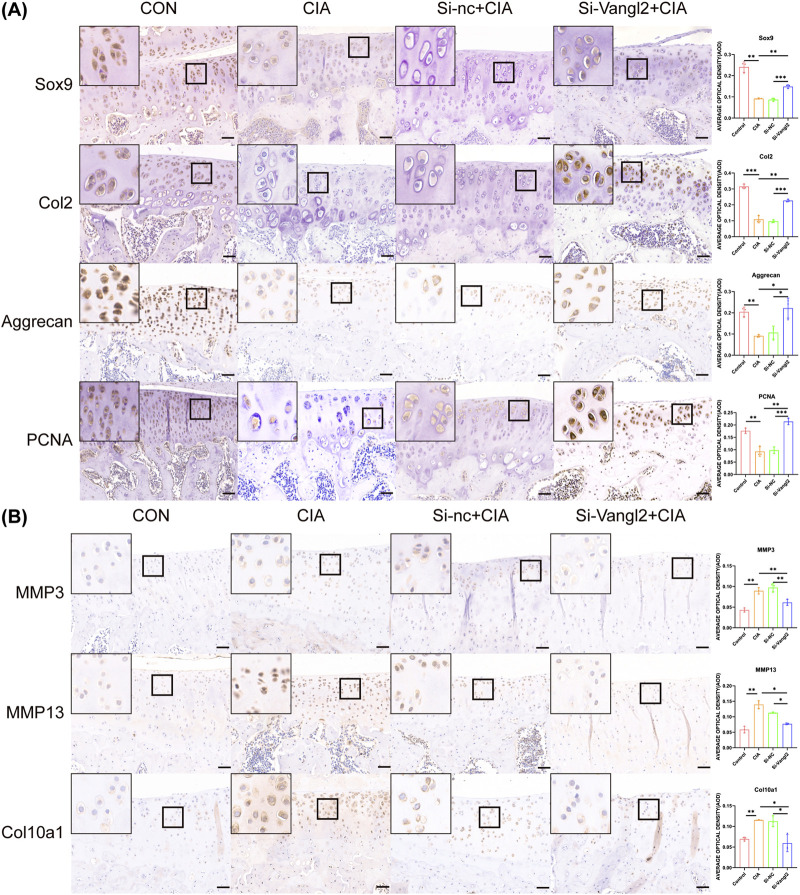
Functional effects of Si-Vangl2 on OA model. **(A)** Si-Vangl2 rescues the expression of chondrogenic and proliferative markers in OA cartilage. Scale bar = 50 μm. **(B)** Si-Vangl2 suppresses matrix degradation and hypertrophic differentiation in OA cartilage. Scale bar = 50 μm. The values are presented as means ± SD (n = 3). *p < 0.05, **p < 0.01, ***p < 0.001.

With the established OA model and the effective intra-articular knockdown of Vangl2, we performed IHC analysis to elucidate the molecular basis of the cartilage-protective effect of Si-Vangl2. IHC analysis showed reduced expression of Sox9, Col2, and Aggrecan in the CIA and the Si-nc + CIA groups. Si-Vangl2 administration increased the expression of these markers in articular cartilage. PCNA expression was fewer in the CIA groups and were increased after Si-Vangl2 treatment (Figure 4A).

### Si-Vangl2 suppressed matrix degradation and hypertrophic differentiation in OA cartilage

Given its chondrogenic and proliferative effects, we subsequently evaluated the impact of Si-Vangl2 on matrix-catabolism and hypertrophy-related markers in OA. The expression of MMP3 and MMP13 were elevated in the CIA and the Si-nc + CIA groups, whereas Si-Vangl2 treatment reduced the expression levels. Col10a1 staining was increased in the CIA and nc groups and was reduced following Si-Vangl2 administration (Figure 4B).

Si-Vangl2 treatment significantly suppressed the aberrant expression of Col10a1 and MMP13, suggesting an inhibitory effect on pathological chondrocyte hypertrophy.

### Si-Vangl2 attenuated the activation of Wnt/PCP-associated JNK and NF-κB signaling in OA cartilage

**FIGURE 5:**
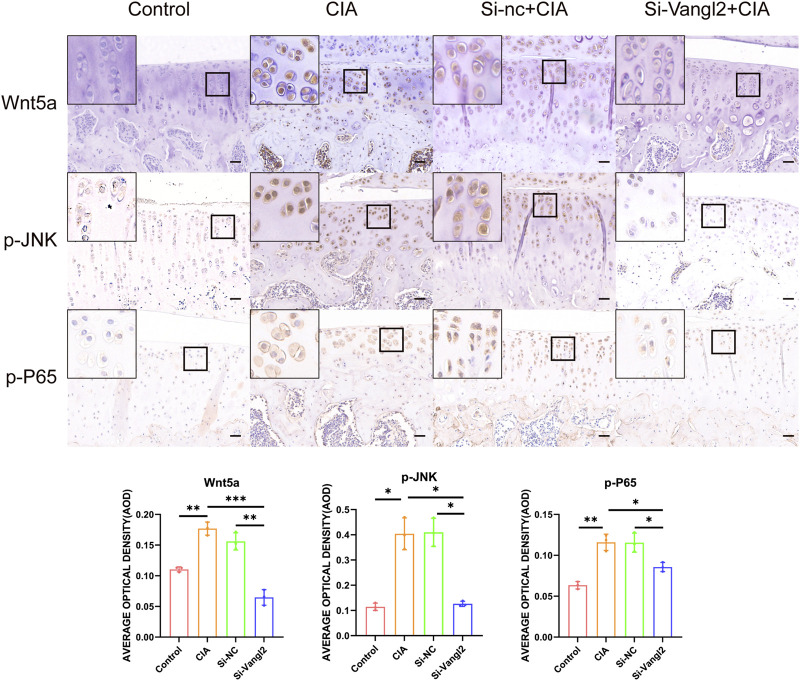
Si-Vangl2 attenuates the activation of Wnt5a associated JNK and NF-κB signaling in OA cartilage. Scale bar = 50 μm. The values are presented as means ± SD (n = 3). *p < 0.05, **p < 0.01, ***p < 0.001.

To further investigate the mechanisms underlying the protective effects of Vangl2 silencing, we examined the expression of Wnt5a, p-JNK, and p-P65 in the CIA and Si-nc + CIA groups. All three markers were notably elevated, indicating activation of JNK and NF-κB signaling under inflammatory and degenerative conditions. In contrast, Si-Vangl2 treatment reduced their staining intensity, demonstrating that Vangl2 silencing attenuates Wnt5a-associated JNK/NF-κB activation *in vivo* (Figure 5).

## Discussion

OA is a prevalent and progressive joint disorder associated with an increasing socioeconomic burden (Martel-Pelletier et al., 2016). Abnormal metabolism in articular chondrocytes is a crux of this multifactorial disease (Mobasheri et al., 2017; Zheng et al., 2021). To our knowledge, this is the first study showing that intra-articular silencing of Vangl2 limits cartilage degeneration *in vivo* by enhancing anabolic activity, suppressing catabolic and hypertrophic pathways, and partially restoring chondrocyte homeostasis.

As a core component of the Wnt/PCP signaling network (Gao et al., 2011) Vangl2 exhibits context-dependent functions in chondrocytes, reflecting microenvironment-dependent signaling reprogramming. Under physiological conditions, Vangl2 is essential for maintaining chondrocyte polarity and growth plate chondrocytes organization (Randall et al., 2012). In this study, Vangl2 supports proliferation and chondrogenic differentiation in ATDC5 (Figure 2). Conversely, in both our *in vivo* CIA-induced OA model and previous *in vitro* inflammatory model (Zhang et al., 2021), Vangl2 was significantly upregulated alongside Wnt5a and JNK/NF-κB activation (Figures 1, 5). Indeed, Vangl2 may facilitate the Wnt5a/PCP-mediated catabolism in OA, forming a pro-inflammatory amplification loop that accelerates matrix degradation (Zhao et al., 2014; Huang et al., 2017; Gill et al., 2023). Such context-dependent functional switching is not unique to Vangl2. Certain members of the transforming growth factor-beta (TGF-β) family, such as TGF-β1 and TGF-β3, similarly exert pleiotropic or even opposing effects on chondrocyte homeostasis depending on the disease stage and local microenvironment (Lin et al., 2022; Du et al., 2023). These observations suggest that Vangl2 becomes aberrantly engaged in inflammatory networks during OA, justifying Vangl2 knockdown as a targeted strategy to restore chondrocyte homeostasis and counteract OA progression.

Notably, a recent high-impact study demonstrated that local administration of a small molecule inhibitor could reprogram hypertrophic-like chondrocytes toward ECM-synthesizing phenotype, thereby improving cartilage structure (Singla et al., 2025). This concept underscores that restoring the balance between matrix synthesis and degradation, together with suppression of pathological hypertrophy, is central to effective disease-modifying strategies for OA (van der Kraan and van den Berg, 2012; Cho et al., 2021; Mazloomnejad et al., 2025).

In line with this emerging concept, intra-articular silencing of Vangl2 restored chondrocyte homeostasis in OA by simultaneously enhancing anabolic and proliferative programs while restraining catabolic and hypertrophic pathways. Si-Vangl2 treatment upregulated Sox9, a key transcription factor driving chondrogenic differentiation (Song and Park, 2020), and its downstream matrix components Col2, Aggrecan, together with increased PCNA expression, indicating enhanced ECM synthesis and chondrocyte proliferation (Figure 4A) (Dong et al., 2023; Sim et al., 2024). In parallel, Vangl2 silencing markedly attenuated ECM catabolism by suppressing the expression of MMP3 and MMP13 (Figure 4B), two major mediators of cartilage matrix degradation in OA (Mehana et al., 2019). MMP13 is primarily responsible for collagen II and Aggrecan cleavage (Chen et al., 2012), while MMP3 degrades proteoglycans and non-fibrillar matrix components and also activates latent pro-MMPs, thereby amplifying the protease cascade that drives ECM destruction (Wan et al., 2021).

Notably, suppression of canonical hypertrophic markers such as Col10a1 and MMP13 suggests partial stabilization of chondrocyte phenotype (Figure 4B). Hypertrophic transition is increasingly recognized as a driver of OA progression (Singh et al., 2019), as it is associated with early cartilage-to-bone shifting, matrix mineralization, and reduced proliferative capacity (Chawla et al., 2022; Han et al., 2024). Thus the observed molecular changes collectively support attenuation of catabolic signaling and degenerative remodeling after Vangl2 silencing.

At the signaling level, phosphorylation of JNK and P65 were markedly reduced *in vivo* following Si-Vangl2 treatment, implicating modulation of Wnt5a-associated inflammatory signaling (Figure 5). Aberrant activation of NF-κB, MAPK, and Wnt signaling is now considered a major pathogenic feature of knee OA (Fazio et al., 2024; Fan et al., 2025). Wnt5a has been shown to activate NF-κB via IκBα degradation and P65 nuclear translocation, while stimulate JNK signaling, amplifying pro-inflammatory cytokines (IL-1β, IL-6) production and ECM catabolism within the articular microenvironment (Zhao et al., 2014; Choi et al., 2019; Cherifi et al., 2021). Importantly, NF-κB signaling also plays a central role in chondrocyte hypertrophy by upregulating hypertrophy-associated genes, including MMP13, Col10a1, Runx2, and VEGF (Choi et al., 2019). Although our previous *in vitro* study demonstrated suppression of multiple MAPK branches following Vangl2 knockdown (Zhang et al., 2021), sustained inhibition of p-JNK was most evident *in vivo*. This discrepancy may reflect distinct temporal dynamics and context-specific activation patterns of MAPK signaling in articular cartilage. JNK signaling, in particular, exhibits sustained activation in OA and has been implicated in AP-1-dependent expression of MMPs and pro-inflammatory cytokines (Han et al., 2001; Johnson and Nakamura, 2007; Xu et al., 2021; Minghua et al., 2024). ERK activation however is transient and associated with proliferative or mechanical responses, which limits the IHC detectability (Xu et al., 2021). Therefore, attenuation of JNK/NF-κB activation likely contributes to the observed reduction in inflammatory and catabolic responses.

**FIGURE 6:**
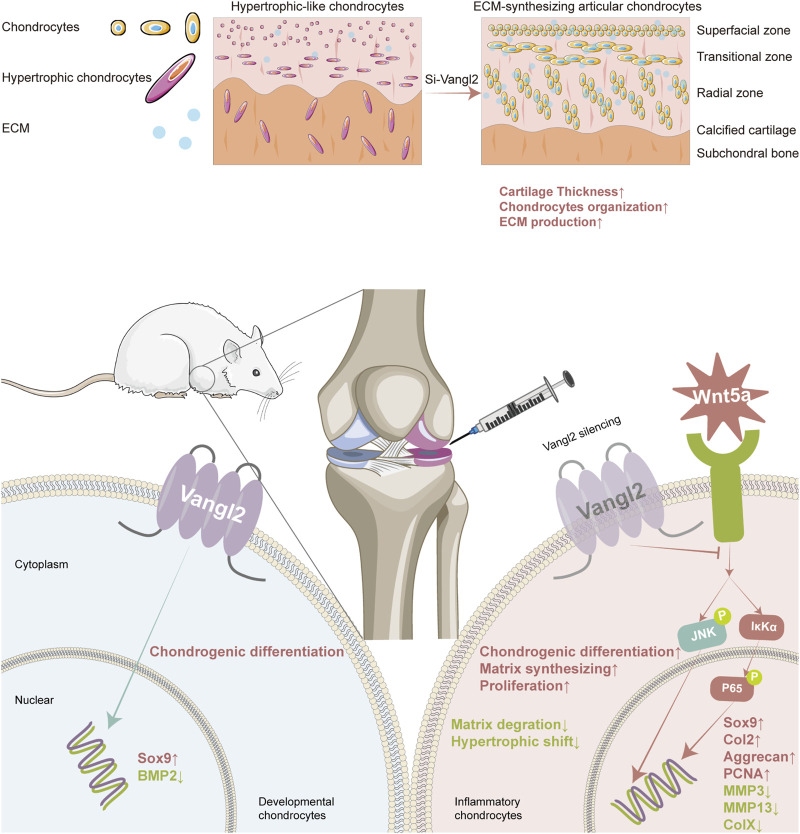
Proposed model of Si-Vangl2 promoted cartilage protection and repair in osteoarthritis. A schematic abstract illustrating intra-articular Vangl2 silencing protects articular cartilage and promotes cartilage repair in osteoarthritis. Under OA conditions, intra-administration of Si-Vangl2 attenuates Wnt5a-associated PCP signaling, suppresses JNK and NF-κB activation, so that chondrogenic differentiation and matrix synthesis are enhanced, catabolic and hypertrophic processes are restrained. At cellular level, hypertrophic-like chondrocytes shift toward an ECM-synthesizing phenotype. As a result, cartilage structure and integrity are partially restored.

Collectively, our findings indicate that Vangl2 functions as a critical factor of chondrocyte homeostasis in OA. Histologically, intra-articular silencing of Vangl2 partially restored cartilage surface integrity, matrix preservation, and chondrocyte organization. At the cellular level, Si-Vangl2 shifted chondrocytes toward an anabolic and proliferative phenotype, accompanied by reduced expression of catabolic enzymes and hypertrophic markers. Mechanistically, Vangl2 silencing attenuated activation of JNK and NF-κB signaling *in vivo*, suggesting suppression of Wnt5a-associated pro-inflammatory pathways. Together, these data support a model in which silencing of Vangl2 confers cartilage-protective effects during OA progression (Figure 6).

OA remains a major unsolved medical challenge, affecting one in three people over the age of 65, with no FDA-approved disease-modifying therapies (Singla et al., 2025). As a locally deliverable RNA-based agent, Si-Vangl2 represents a potential strategy for targeted modulation of joint inflammatory signaling. However, several limitations should be acknowledged. The long-term therapeutic efficacy and pharmacokinetics of intra-articular Si-Vangl2 were not evaluated in the present study. Further studies are warranted to validate and optimize its long-term efficacy, safety and translational potential of Vangl2-targeted therapy in OA.

## Conclusion

For the first time, this study demonstrates that intra-articular delivery of Si-Vangl2 alleviates cartilage degeneration in experimental OA. Si-Vangl2 enhances chondrocyte proliferation and anabolic activity while suppressing matrix degradation and pathological hypertrophic differentiation, by attenuates Wnt5a associated JNK/NF-κB pathway, highlighting Vangl2 as a potential disease-modifying target for OA treatment.

## Data Availability

The datasets presented in this study can be found in online repositories. The names of the repository/repositories and accession number(s) can be found in the article/Supplementary Material.
