# Supplementary figures and images for: Intra-articular delivery of Si-Vangl2 limits cartilage degeneration in an osteoarthritis rat model
Source: Front Bioeng Biotechnol. 2026 Mar 30;14:1773841. doi: 10.3389/fbioe.2026.1773841 (PMC13071021; doi:10.3389/fbioe.2026.1773841)

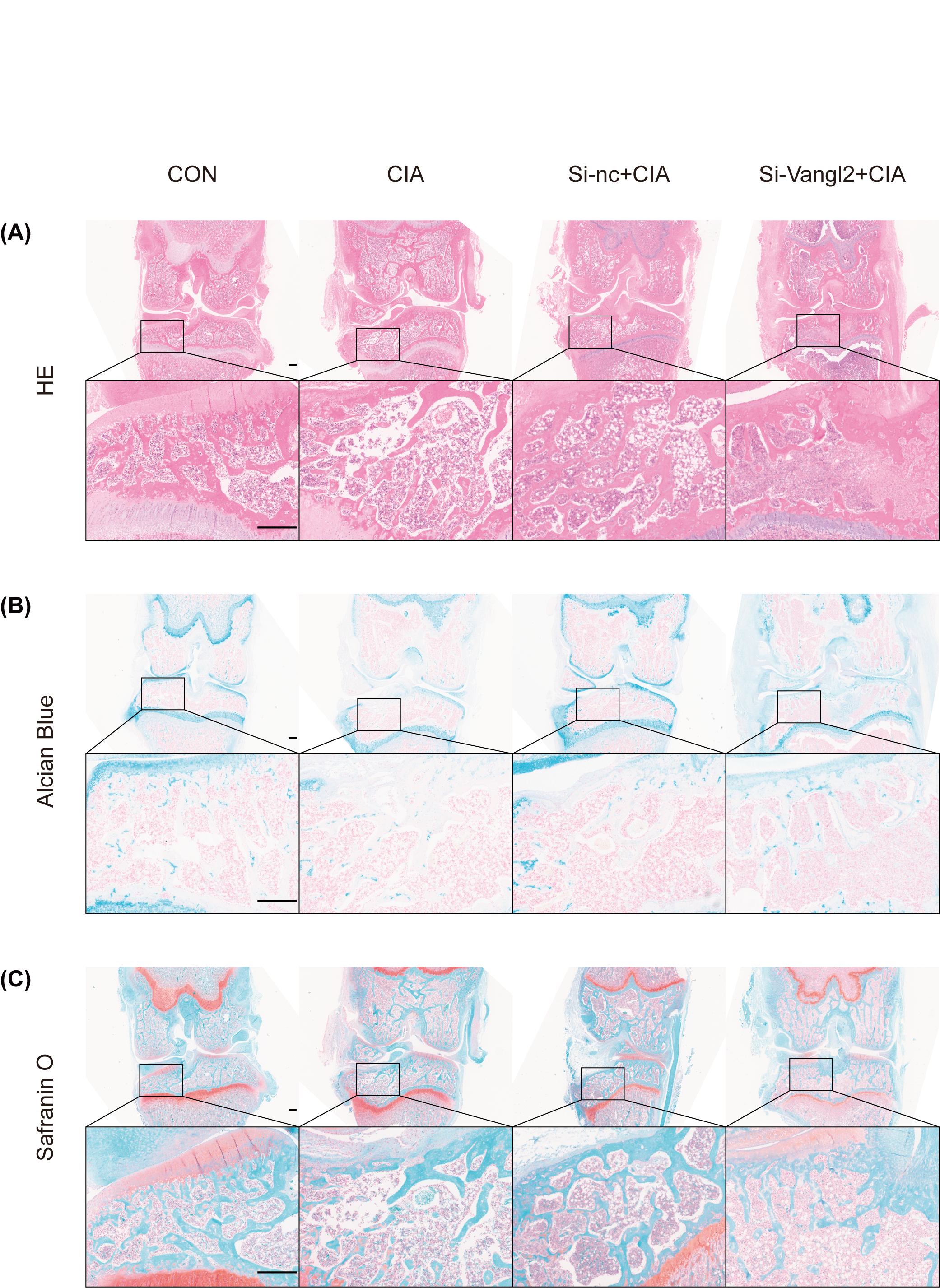

Supplement: Supplementary file 1 [file Image2.tif]

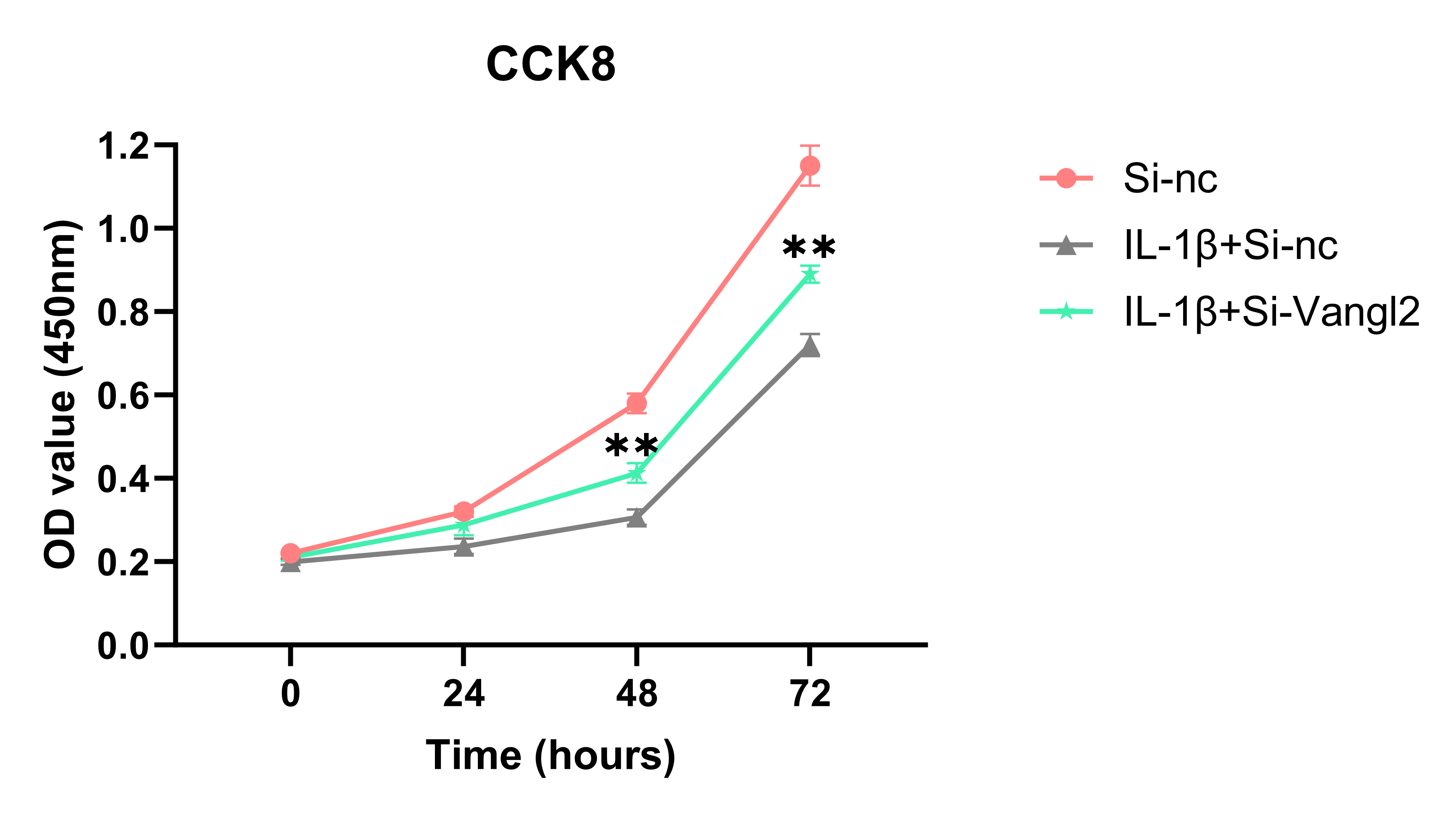

Supplement: Supplementary file 2 [file Image1.tif]
